# Unveiling regional differences in glioblastoma patient survival with real-world data from the Norwegian brain tumor quality registry

**DOI:** 10.1007/s11060-025-05218-3

**Published:** 2025-09-11

**Authors:** Cassia Bree Trewin-Nybråten, Paul Christopher Lambert, Kirsten Marienhagen, Lasse Andreassen, Tom Børge Johannesen, Pitt Niehusmann, Leif Oltedal, Stephanie Schipmann, Anne Jarstein Skjulsvik, Ole Solheim, Tora Skeidsvoll Solheim, Terje Sundstrøm, Einar Osland Vik-Mo, Petter Brandal, Tor Ingebrigtsen, Erlend Skaga

**Affiliations:** 1 Department of Registration, Cancer Registry of Norway, Norwegian Institute of Public Health, Oslo, Norway; 2 Department of Medical Epidemiology and Biostatistics, Karolinska Institutet, Stockholm, Sweden; 3 Department of Oncology, University Hospital of North Norway, Tromsø, Norway; 4 Department of Neurosurgery, Otorhinolaryngology and Ophthalmology, University Hospital of North Norway, Tromsø, Norway; 5 Department of Pathology, Oslo University Hospital, Oslo, Norway; 6 Division for Cancer Medicine, Oslo University Hospital, Oslo, Norway; 7 Mohn Medical Imaging and Visualization Centre, Department of Radiology, Haukeland University Hospital, Bergen, Norway; 8 Department of Clinical Medicine, University of Bergen, Bergen, Norway; 9 Department of Neurosurgery, Haukeland University Hospital, Bergen, Norway; 10 Department of Neurosurgery, University Hospital Muenster, Münster, Germany; 11 Department of Pathology, St. Olavs University Hospital, Trondheim, Norway; 12 Department of Clinical and Molecular Medicine, Faculty of Medicine and Health Sciences, Norwegian University of Science and Technology, Trondheim, Norway; 13 Department of Neurosurgery, St. Olavs University Hospital, Trondheim, Norway; 14 Department of Neuromedicine and Movement Science, Norwegian University of Science and Technology, Trondheim, Norway; 15 Cancer Clinic, St. Olavs University Hospital, Trondheim, Norway; 16 Vilhelm Magnus Laboratory for Neurosurgical Research, Oslo University Hospital, Oslo, Norway; 17 Department of Neurosurgery, Oslo University Hospital, Oslo, Norway; 18 Institute for Clinical Medicine, Faculty of Medicine, University of Oslo, Oslo, Norway; 19 Department of Oncology, Division of Cancer Medicine, Oslo University Hospital, Oslo, Norway; 20 Institute for Cancer Genetics and Informatics, Oslo University Hospital, Oslo, Norway; 21 Department of Clinical Medicine, Faculty of Health Sciences, UiT the Arctic University of Norway, Tromsø, Norway

**Keywords:** Glioblastoma, Survival, Anti-neoplastic treatment, Cancer registries, Regional differences, Norway

## Abstract

**Purpose:**

Surveillance of patient outcomes with real-world data is essential to uncover regional disparities in clinical practice or quality of care. This study explored survival differences among glioblastoma patients in Norway and investigated the role of demographic and treatment factors.

**Methods:**

We analyzed real-world data from the Norwegian Cancer Registry on 1158 adults with histologically confirmed glioblastoma during 2019–2023. Surgical treatment rates per 100,000 inhabitants per region (South-East, West, Mid, North) were compared using adjusted Poisson models. Full treatment included surgical resection, radiotherapy (≥ 55 Gy for ≤ 70 years; ≥30 Gy for > 70 years), and temozolomide. Standardized survival was estimated with flexible parametric models, standardized for age, sex, year, and distances to treatment facilities.

**Results:**

Patients from the North were older and lived further from treatment centers. For patients aged 18–70, treatment and survival did not significantly differ across regions; national median standardized survival was 14.4 months (95%CI:13.6–15.2). For elderly patients (71–89 years), the North demonstrated a higher surgical treatment rate (rate ratio = 1.32; 95%CI = 0.99–1.77), but lesser use of postoperative radiotherapy and temozolomide. Median standardized survival for elderly patients in the North was 4.5 months (95%CI: 3.5–5.7) versus 7.7 (6.9–8.6) months nationally. Early mortality was particularly high for elderly patients in the North, yet those surviving beyond six months matched other regions’ survival probability.

**Conclusion:**

Lower glioblastoma survival in the North was associated with higher early mortality among elderly patients, likely due to selecting frailer patients for surgery, who less often subsequently received anti-neoplastic treatment.

**Supplementary Information:**

The online version contains supplementary material available at 10.1007/s11060-025-05218-3.

## Introduction

Real-world data is crucial for surveilling patient outcomes, evaluating healthcare effectiveness and improving equity of care [1–3]. Real-world data from the Norwegian Brain and Spinal Cord Tumour Registry (NBTR) has revealed regional discrepancies in survival of glioblastoma patients, with poorest outcomes for patients residing in North Norway during 2018–2022 [4]. Norway has a universal healthcare system designed to ensure equal access to health services. Glioblastoma treatment is centralized to regional centers that follow national treatment guidelines [5]. Thus, regional disparities in glioblastoma survival raise concerns about potential variations in practice or quality of cancer care.

Glioblastoma has aggressive behavior, limited treatment options and poor survival outcomes [6]. The standard of care has remained unchanged for the past two decades, consisting of maximum safe surgical resection, 60 Gy of radiotherapy and concomitant and adjuvant temozolomide [7]. Patients treated with this multimodal approach have around 15 months median survival and below 30% two-year survival probability [7, 8]. However, not all patients are candidates for standard treatment [9]; and the median survival in unselected populations is around 12 months, with a two-year survival probability below 20% [10, 11]. Tumor characteristics like eloquent localization or multifocality might limit the feasibility of surgical resection, while patient factors like functional status, comorbidity or advanced age may preclude suitability for surgery, full radiotherapy or chemotherapy. Travel distance to treatment facilities, which can be far in the Northern region, may also affect treatment decisions [12]. For patients over 70 years, hypofractionated radiotherapy up to 40 Gy is recommended [13, 14]. The frailest patients who are considered unsuitable for anti-neoplastic treatment may or may not undergo diagnostic surgery. Type of surgery, radiation dose and temozolomide treatment, as well as age – all independently influence patient survival [15, 16].

Determining the factors responsible for regional survival variation is essential for addressing these disparities. We utilized nationwide real-word data from the NBTR – including prospectively collected patient, tumor and treatment data, to quantify regional differences in glioblastoma patient survival and explore potential associations with demographic factors and treatment patterns.

## Materials and methods

### Design and data

This nationwide registry-based study utilized data from the Norwegian Brain and Spinal Cord Tumour Registry (NBTR), which was integrated with the Cancer Registry of Norway (CRN) in 2022 and included all previously collected data on brain and spinal cord tumors in the CRN. Since 1953, the CRN has had mandatory reporting of new cancer cases and is considered highly complete and valid [17]. A recent study estimated 98.8% completeness nationally for all new glioblastoma cases in the CRN during 2002–2021 [11]. Histologically confirmed cases are practically complete, whereas clinically diagnosed cases that remain untreated may be underreported (Online Resource 1).

## Study population

In the CRN, we identified 1230 adult cases of histologically confirmed glioblastoma diagnosed between January 1, 2019, and December 31, 2023 (Online Resource 1). Glioblastoma was defined by the International Classification of Diseases, Oncology 3rd Edition, with topography C71–C72 and histopathology 9440–2 (2016 WHO classification). IDH-status was not available. Of 1230 registered cases, we excluded 2 patients that were > 89 years at diagnosis, 11 (1%) with an unknown residential region, and 59 (5%) that were diagnosed after September 30, 2023, due to insufficient radiotherapy data. Data on dispensed temozolomide was available until June 17, 2024. Our final study population included 1158 patients, followed from histological diagnosis until death, emigration, two years follow-up, or September 30, 2024, whichever came first.

## Regional health authorities

Norway is organized into four regional health authorities, hereby referred to as regions, that were responsible for patient care, including transportation to and from treatment facilities within that defined geographical area. Patients are routinely referred to treatment within their region of residence; however, 19 patients in the study population (1.6%) received treatment outside their registered region of residence and were categorized by where they were treated.

## Demographic characteristics

**Fig. 1:**
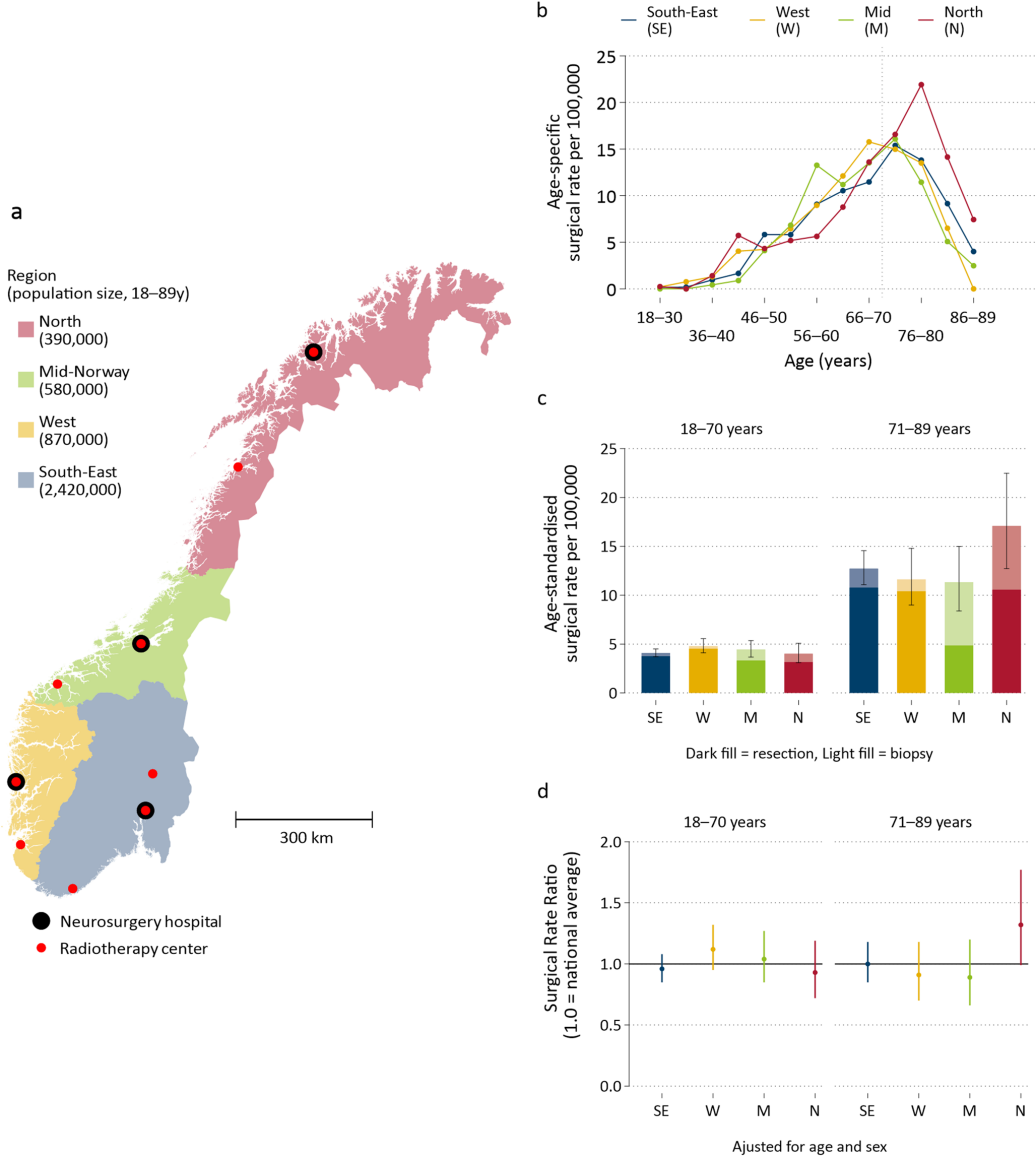
Regional differences in rate of histologically confirmed glioblastoma per 100,000 inhabitants aged 18–89 years, 2019–2023. **a** Regions and their population size; **b** Age-specific surgical rate; **c** Age-standardized surgical resection and biopsy rates; and **d** Poisson rate ratios, adjusted for age and sex (*N* = 1158 cases; 21 million person-years)

Characteristics included sex, age, year and residential municipality, categorized by quartile of distance from the municipality midpoint to the region’s closest neurosurgical hospital and radiotherapy center (Online Resource 2). Each region was served by one neurosurgical hospital conducting brain tumor surgery and two or three radiotherapy centers (Fig. 1A). Thus, the quartile cutoffs were longer for surgical hospitals (Q2: >68 km, Q3: >128 km, Q4: >211 km) than for radiotherapy centers (Q2: >43 km, Q3: >74 km, Q4: >118 km).

## Treatment variables

Surgery was categorized as resection (partial or complete) or biopsy. Assuming glioblastoma incidence was similar across regions, surgical treatment rate was defined as the incidence of histologically confirmed glioblastoma per 100,000 inhabitants aged 18–89 years in each region. Completed radiation was defined as ≥ 55 Gy for patients aged 18–70 years and ≥ 30 Gy for patients aged 71–89 years at diagnosis. Patients were classified as receiving temozolomide if at least one prescription was dispensed after surgery. The Prescription Database did not distinguish between concomitant and adjuvant temozolomide use.

Combined treatment with surgery, radiotherapy and temozolomide was categorized separately by patient’s age (18–70 or 71–89 years), reflecting differences in current clinical practice by age [14]. Complete treatment for patients aged 18–70 years was surgical resection, ≥ 55 Gy radiation and temozolomide. For patients aged 71–89 years, any surgery type, ≥ 30 Gy radiation and temozolomide was considered complete treatment.

### Statistical analysis

Kaplan-Meier estimates of overall survival were plotted by region and compared with a log-rank test. Distribution of demographic and treatment variables were compared between regions with chi-squared tests. Median age and crude median overall survival were estimated for covariates.

To compare surgical rates, we plotted age-standardized rates of histologically confirmed glioblastoma per 100,000 inhabitants per region. Using Poisson regression, we then estimated incidence rate ratios (IRR) with 95% confidence intervals (CI) adjusted for age, sex and year for each region versus the national average.

Using flexible parametric survival models [18, 19], we compared regions for all-cause mortality. The final model included region, age, sex, year, distance to surgical hospital and radiotherapy center, and had a two-way interaction between region and non-linear age and a three-way interaction between region, non-linear age and time since diagnosis. Non-linear age was modelled with natural splines with three degrees of freedom and winsorized at the 5th and 95th percentiles due to sparse data at the youngest and oldest ages. All-cause mortality rate ratios with 95% CI were estimated for each region versus the national average and plotted by time since diagnosis. We estimated p-values from likelihood ratio tests comparing models with and without region.

From flexible parametric survival models, we obtained adjusted survival curves comparing regions with regression standardization. These estimate the survival that would have been observed if all regions had the same distribution of variables included in the model [20]. For the age group 18–70 years (covered by national treatment guidelines) and 71–89 years, we estimated standardized median, one-year and two-year survival and difference in survival for each region compared to the national average.

The analyses were conducted in Stata-18 [21]. A two-sided *P* < 0.05 was considered statistically significant. The statistics were produced in-house at CRN, which is statutory, so consent was not required according to the Norwegian Health Register Act § 19.

## Results

### Patient characteristics

The 1158 patients with a histologically confirmed glioblastoma had a median age of 65 years at diagnosis and median overall survival of 12.2 months (95% CI: 11.4–12.8) (Table 1). Patients from the North region, representing 10% (*N* = 117) of the cohort, had a median age of 69 years compared to 64–65 years in other regions. Patients in the North also lived more remotely, which was associated with poorer overall survival than living in the closest quartile of distance to treatment centers (Table 1, Online Resource 3). The sex distribution was similar across regions with a slight male predominance (ratio 1.5:1). Use of surgical resection varied by region; 65–70% of patients who underwent surgery in the Mid and North regions had a tumor resection, compared to > 90% of patients in the South-East and West. Additionally, the North region had the lowest proportion of patients who received radiotherapy or temozolomide, whereas the Mid-region had the highest. Overall survival according to age-specific treatment groups is presented in Online Resource 4.

**Table 1:** Patient and treatment characteristics by region. Adults with histologically confirmed glioblastoma at 18–89 years, 2019–2023 (*N* = 1158)

			Region					Median age years (SD)	Median overall survival months (95% CI)
Variable	Total *N* (%)		South-East *N* (%)	West *N* (%)	Mid *N* (%)	North *N* (%)	Chi^2^ *P*		
Total	1158 (100)		631 (100)	242 (100)	168 (100)	117 (100)		65 (12)	12.2 (11.4–12.8)
Sex							0.366		
Male	689 (59.5)		366 (58.0)	156 (64.5)	98 (58.3)	69 (59.0)		64 (12)	11.6 (11.0–12.4)
Female	469 (40.5)		265 (42.0)	86 (35.5)	70 (41.7)	48 (41.0)		65 (11)	13.0 (12.0–13.7)
Age at diagnosis							0.032		
*Median (SD)*	*65 (12)*		*64 (12)*	*64 (12)*	*65 (10)*	*69 (12)*			
18–70 years	778 (67.2)		420 (66.6)	176 (72.7)	115 (68.5)	67 (57.3)		59 (9)	14.2 (13.4–14.9)
71–89 years	380 (32.8)		211 (33.4)	66 (27.3)	53 (31.5)	50 (42.7)		75 (4)	7.4 (6.3–8.4)
Distance to neurosurgical hospital, quartile (Q)							< 0.001		
Q1 (≤ 68 km)	569 (49.1)		358 (56.7)	106 (43.8)	83 (49.4)	22 (18.8)		64 (11)	13.5 (12.6–14.5)
Q2 (69–128 km)	224 (19.3)		165 (26.1)	32 (13.2)	20 (11.9)	7 (6.0)		65 (11)	10.4 (8.3–12.1)
Q3 (129–211 km)	203 (17.5)		51 (8.1)	98 (40.5)	28 (16.7)	26 (22.2)		66 (12)	11.4 (9.7–12.6)
Q4 (212–586 km)	162 (14.0)		57 (9.0)	6 (2.5)	37 (22.0)	62 (53.0)		65 (12)	10.8 (8.9–12.4)
Distance to radiotherapy center, quartile (Q)					< 0.001				
Q1 (≤ 43 km)	676 (58.4)		367 (58.2)	173 (71.5)	102 (60.7)	34 (29.1)		64 (12)	13.0 (12.2–13.8)
Q2 (44–74 km)	208 (18.0)		127 (20.1)	39 (16.1)	24 (14.3)	18 (15.4)		66 (11)	11.5 (10.0–12.7)
Q3 (75–118 km)	173 (14.9)		126 (20.0)	6 (2.5)	21 (12.5)	20 (17.1)		65 (11)	10.8 (8.5–13.3)
Q4 (119–468 km)	101 (8.7)		11 (1.7)	24 (9.9)	21 (12.5)	45 (38.5)		69 (13)	9.4 (8.6–13.2)
Type of surgery							< 0.001		
Resection	989 (85.4)		571 (90.5)	225 (93.0)	109 (64.9)	84 (71.8)		64 (12)	13.2 (12.5–13.8)
Biopsy	169 (14.6)		60 (9.5)	17 (7.0)	59 (35.1)	33 (28.2)		71 (10)	5.3 (3.9–6.3)lePara>
Radiotherapy							0.002		
Yes (any dose)	1012 (87.4)		544 (86.2)	217 (89.7)	158 (94.0)	93 (79.5)		64 (11)	13.4 (12.9–14.2)
No	146 (12.6)		87 (13.8)	25 (10.3)	10 (6.0)	24 (20.5)		72.5 (11)	2.0 (1.5–2.6)
Temozolomide							< 0.001		
Yes	964 (83.2)		530 (84.0)	197 (81.4)	154 (91.7)	83 (70.9)		63 (11)	14.0 (13.3–14.6)
No	194 (16.8)		101 (16.0)	45 (18.6)	14 (8.3)	34 (29.1)		73 (10)	2.6 (2.2–3.3)

### Rate of histologically confirmed glioblastoma

Up to age 70 years, the surgical rate per 100,000 inhabitants was consistent across all regions, as was median age at diagnosis (59–60 years). However, for the older age group (71–89 years), the surgical rate per 100,000 inhabitants – particularly biopsy rate – was higher in the North than in other regions (Fig. 1b, c), with a rate ratio of 1.32 (95% CI: 0.99–1.77; *p* = 0.06) compared to the national average (Fig. 1d). In the older age group, the median age at diagnosis was 77 years in the North versus 75 years in other regions.

### Overall survival and adjusted survival

**Fig. 2:**
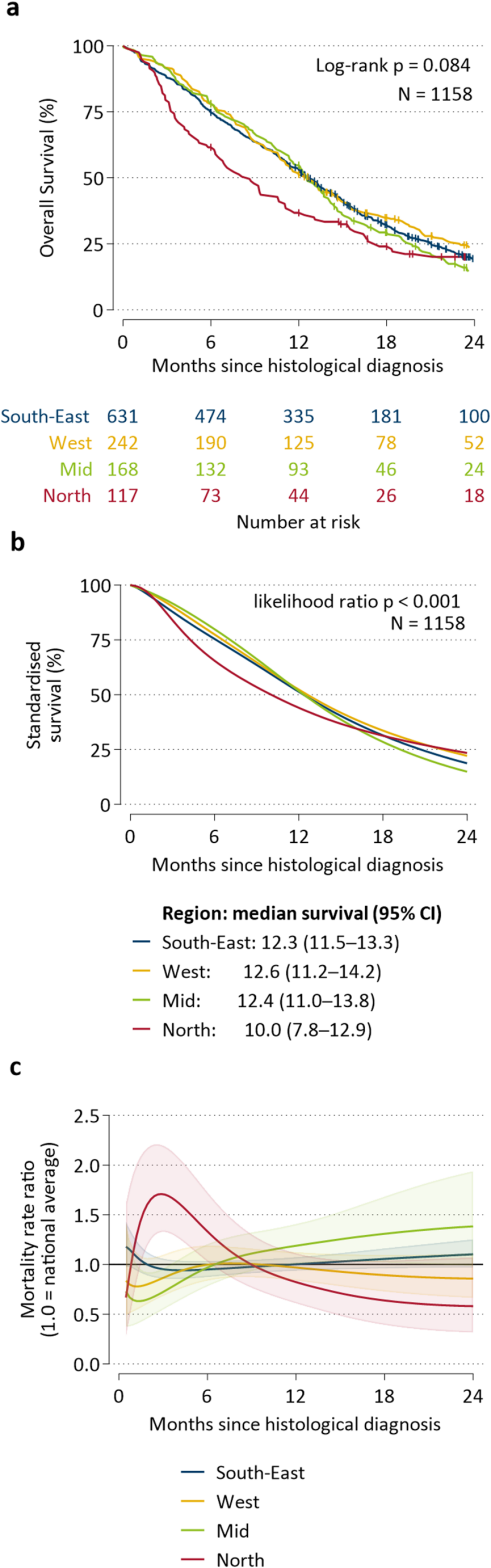
Regional differences in crude and standardized glioblastoma survival; **a** Kaplan-Meier overall survival; **b** Standardized survival^a^ and likelihood ratio test p-value; and **c** hazard ratio^a^ by time since diagnosis. 18–89 years, 2019–2023 (*N* = 1158). ^a^Adjusted for age, sex, year and distance to neurosurgical hospital and radiotherapy center

Patients from the North had the lowest crude overall survival (Fig. 2a). Despite patients in the North living more remotely, adjusting for distance to surgical hospital and radiotherapy center, as well as age, sex and year, had little impact on the estimated survival differences between regions. Standardized survival remained lowest in the North, with a median standardized survival of 10 months, compared to over 12 months in other regions (Fig. 2b). Patients residing in the North had significantly higher mortality during the first six months after diagnosis compared to the national average (Fig. 2c) and the highest 90-day post-operative mortality of 21% versus 8–12% in other regions (Online Resource 5).

### Age-specific treatment patterns and overall survival

**Fig. 3:**
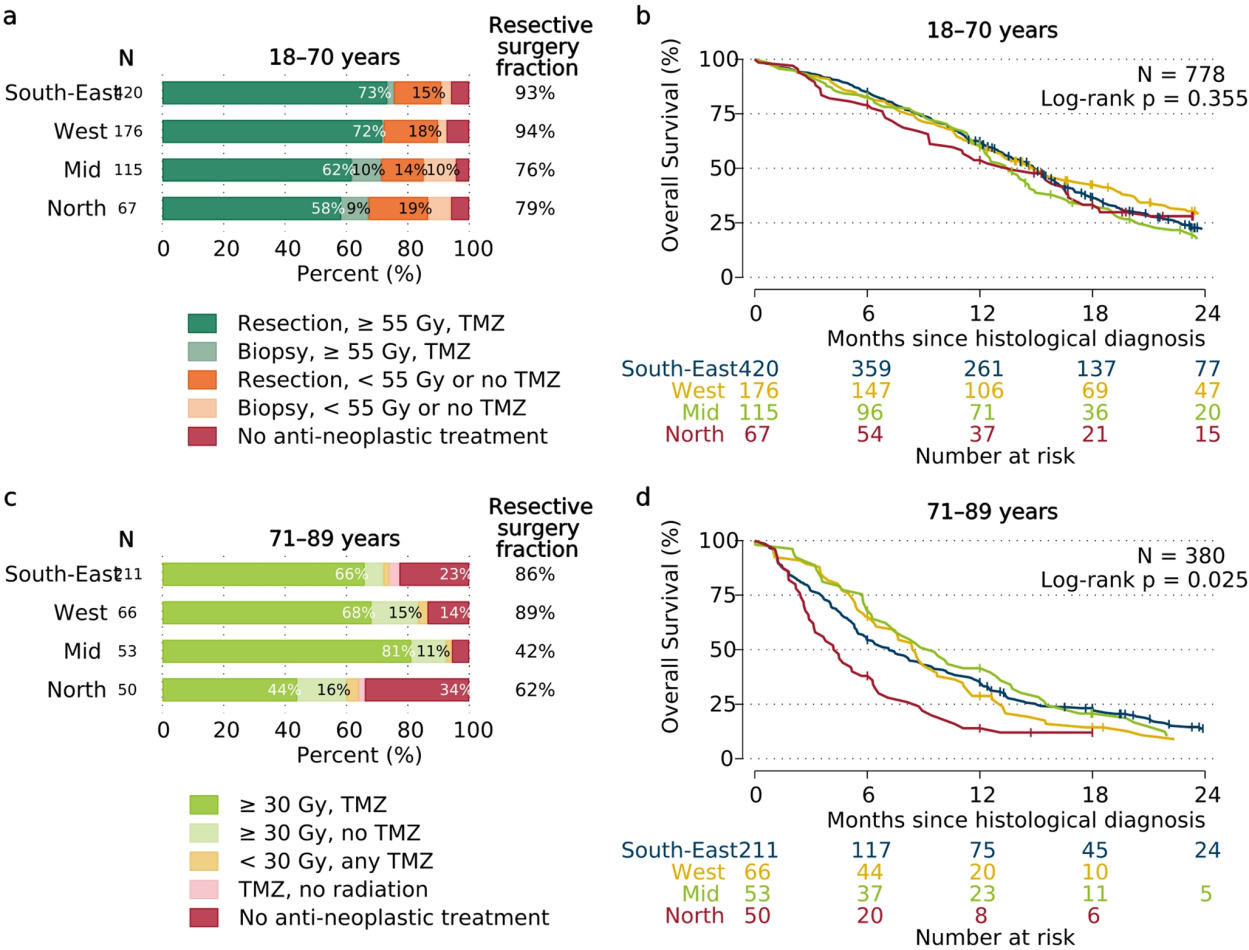
Regional differences in age-specific treatment^a^ and glioblastoma survival. **a** Treatment and **b** overall survival of patients aged 18–70 years (*N* = 778); and **c** treatment and **d** overall survival of patients aged 71–89 years (*N* = 380). ^a^Age-specific current standard-of-care: 18–70 years: maximal safe surgical resection, 60 Gy radiation and temozolomide (TMZ); >70 years: reduced dose short-course radiation with or without TMZ.

In patients aged 18–70 years, surgical resection was chosen over biopsy more frequently in the South-East and West regions (≥ 93% of surgically treated patients) compared to the Mid and North (≤ 79% of patients). Anti-neoplastic treatment differences were more modest; 67% of patients in the North received ≥ 55 Gy radiation combined with temozolomide compared to 71–75% in the other regions (Fig. 3a), and overall survival did not differ significantly between the regions among patients aged up to 70 years (Fig. 3b).

For patients older than 70 years, anti-neoplastic treatment disparities were more pronounced; 60% of patients in the North received ≥ 30 Gy of radiation versus 72–92% in other regions, whereas 46% of patients in the North were treated with temozolomide (alone or with radiation) versus 70–83% in other regions. The Northern region additionally had the largest proportion of patients (34%) who received no anti-neoplastic treatment after surgery (Fig. 3c). Elderly patients in the North also exhibited the lowest overall survival (Fig. 3d).

### Age-specific median survival

**Fig. 4:**
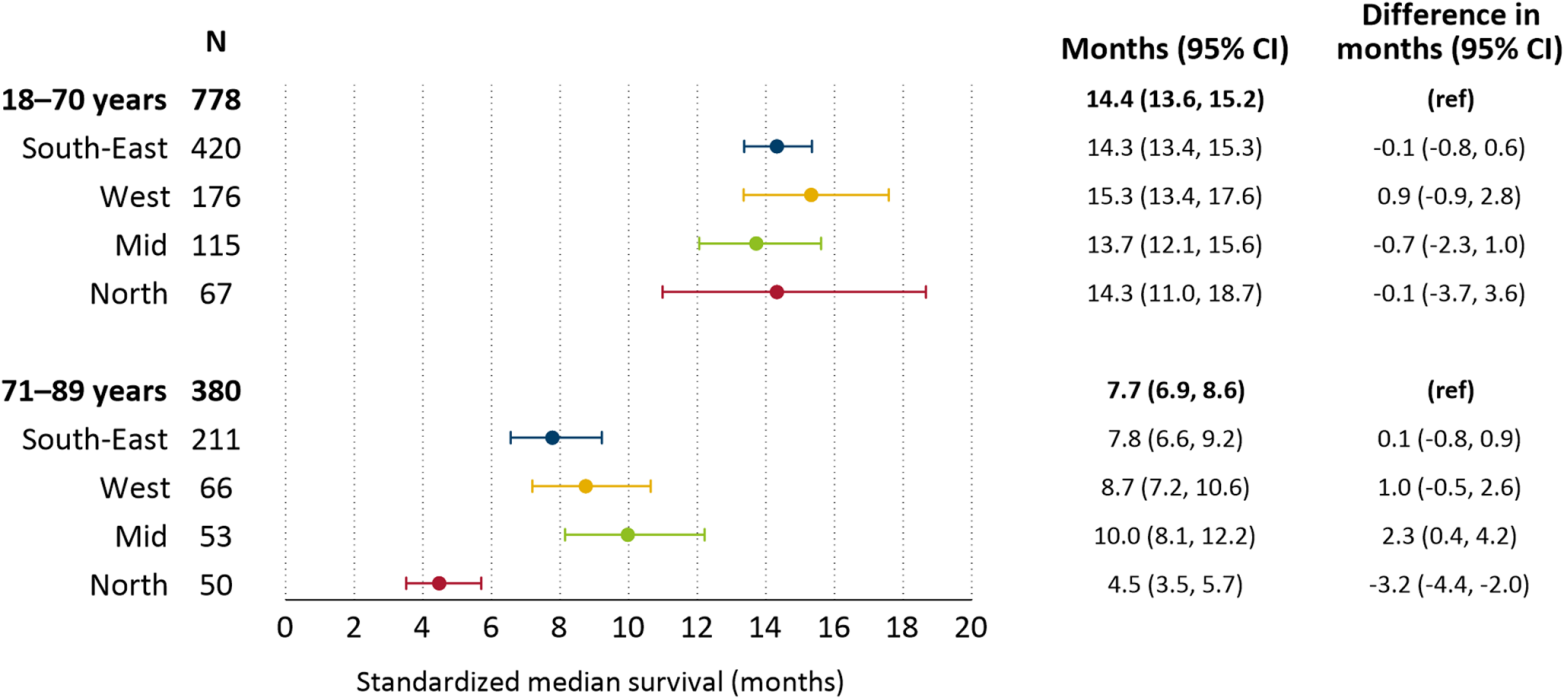
Regional differences in age-specific standardized median survival^a^ from histologically confirmed glioblastoma, 18–89 years, 2019–2023 (*N* = 1158). ^a^Adjusted for age, sex, year and distance to neurosurgical hospital and radiotherapy center

After adjusting for demographic factors, standardized median survival was consistent across regions for patients aged 18–70 years, with a national median survival of 14.4 months (95% CI: 13.7–15.3) (Fig. 4). In this age group, one- and two-year standardized survival was 60% and 23%, respectively (Online Resource 6). Among patients over 70 years, median standardized survival in the North was 4.5 months (95% CI: 3.5–5.7), compared to the national average of 7.7 months (95% CI: 6.9–8.6).

### Six-month conditional survival

Given the significantly raised mortality in the North during the first 6 months after surgery, we finally performed a supplementary analysis of conditional standardized survival of 870 patients aged 18–89 years that survived at least six months after diagnosis. We found no regional survival differences after adjusting for age, sex, year and distance to treatment centers (Online Resource 7). Further adjustment for treatment (type of surgery, radiotherapy and temozolomide) made little difference because treatment patterns were similar by region among patients who had survived long enough to receive radiation and chemotherapy (Online Resource 7).

## Discussion

Our real-world data revealed that compared to other regions, the North had: (i) higher rates of surgical procedures, in particular biopsies, on elderly glioblastoma patients, (ii) a higher proportion of elderly glioblastoma patients not receiving any anti-neoplastic treatment after surgery, and (iii) a significantly higher mortality of glioblastoma patients during the first six months after histological diagnosis. Resultingly, median standardized survival in patients over 70 years was just 4.5 months in the North, compared to 7.7 months nationally. Patients up to 70 years of age had a similar median standardized survival across regions.

Describing disparities in healthcare outcomes and identifying factors associated with these variations are the first steps in addressing health-care inequities. To truly impact healthcare delivery, findings must be contextualized in a manner that transcends traditional epidemiological measures such as risk estimates and ratios – approaches commonly utilized when comparing outcomes across regions or hospitals [22–28]. By integrating high-quality, prospectively collected real-world data with advanced survival analysis, our study quantified survival differences in a clinically meaningful way, while accounting for demographic differences [20]. Specifically, we utilized median survival metrics – typically used in clinical trials [8] – to interpret our results within a clinical context. We demonstrated that median survival time for elderly patients undergoing surgical procedures was three months shorter in the North compared to the national average. More importantly, our methodology highlights the power of comprehensive national registries to expose variations in practice across healthcare settings, providing insights into potential reasons for survival disparities.

The notably higher rate of surgical interventions in patients over 70 years in the North relative to other regions was a key finding. This observation, coupled with high early mortality, is suggestive of a lower threshold for opting for invasive procedures. As many as one in three elderly patients in the North did not receive any anti-neoplastic treatment following surgery. Although it is unclear whether this represented a selection bias of overly frail patients for surgery or a tendency to withhold anti-neoplastic treatment in the elderly after surgery in this region, we believe the former was most probable. Specifically, that elderly patients in the North may have been selected for surgery, then later deemed too frail for further anti-neoplastic treatment. Such variations in the surgical rate among elderly patients may reflect regional differences in the acceptance of radiological diagnoses alone for clinical decision making, thus leading to different thresholds for undertaking invasive procedures when the potential benefit of a definitive histopathological diagnosis is uncertain.

We observed significant regional variations in the type of surgery performed in elderly patients. For instance, while nearly 90% of patients over 70 years underwent tumor resection in the West, only about 40% did so in the Mid region. Although the Mid region had a more conservative surgical approach for elderly patients, they administered anti-neoplastic treatment to nearly all elderly patients, and survival outcomes were on par with the West. The disparities among elderly patients underscore two controversial aspects of glioblastoma management: first, the decision-making process regarding which patients over 70 should undergo surgery for suspected glioblastoma, and secondly, the choice of surgical approach – whether to perform a diagnostic biopsy or proceed with resection.

Criteria for determining whether elderly glioblastoma patients should undergo surgery are not well-defined. Given that age, performance status and anti-neoplastic treatment intensity (radiation and temozolomide) are established prognostic factors for survival in elderly glioblastoma patients [14, 29], those selected for surgery should ideally possess a good performance status (e.g., KPS ≥ 60) and be considered candidates for hypofractionated radiation, temozolomide or both. Our findings show that one out of three patients in the North did not receive any treatment post-surgery highlights an opportunity for improved patient selection. Considering the limited survival benefits of resective surgery alone in the elderly [30], minimizing unnecessary procedures is crucial to avoid undue costs and complications. For context, a large US registry-based report including over 100,000 glioblastoma patients diagnosed 1988–2011 noted that 20.6% of patients aged 75 or older received no post-surgical treatment [31]. In a recent Austrian report, 35% of patients over 70 years with histologically proven glioblastoma did not receive post-operative radiation [32]. Similarly, a study from the Dutch Quality Registry for Neurosurgery reported that 40% of glioblastoma patients who underwent a biopsy during 2011–2021 received no anti-neoplastic treatment [33]. Given the median overall survival for patients receiving no post-operative treatment was 1.8 months in the present study, the NBTR medical council has proposed limiting the fraction of elderly patients undergoing surgery without subsequent anti-neoplastic treatment to below 10% as a quality benchmark.

Current guidelines recommend maximal safe resection for newly diagnosed glioblastoma [34], yet the surgical approach for elderly patients particularly remains contentious. Only two small studies have randomized older patients with suspected high-grade glioma or glioblastoma to undergo biopsy or resection and found little or no survival differences [35, 36]. A population-based Dutch Quality Registry study [33] reported substantial between-hospital variation in biopsy versus resection frequencies, but no notable differences in survival across centers. Despite these findings, resection is often advocated in academic literature, supported mainly by observational data in retrospective series [37].

The major strength of this study was the comprehensive high-quality individual pathology, surgical and anti-neoplastic treatment data [11, 17]. Nevertheless, the study had some shortcomings. Notably, we lacked methylation status of the MGMT promoter, which predicts response to temozolomide [38], as well as performance status, comorbidity, and patient preferences. Further, we couldn’t estimate the true fraction of glioblastoma patients without histopathological confirmation or know whether this varied between regions. A US study [31] reported that 14.3% of 100,672 patients with presumed glioblastoma did not undergo surgery, whereas two recent Norwegian studies [10, 11] estimated that just 5–8% of patients were diagnosed with glioblastoma without tissue confirmation.

## Conclusions

The lower survival among glioblastoma patients in the North region was due to higher mortality among elderly patients within the first six months after surgery. Our real-world data suggested that this was due to a greater selection of elderly patients for invasive procedures, who less often subsequently received anti-neoplastic treatment, compared to other regions. Given the modest benefits of surgery alone for glioblastoma patients over 70 years, reducing the fraction of patients undergoing surgical intervention without subsequent anti-neoplastic treatment should be a priority to avoid unnecessary risk, morbidity and healthcare costs.

## Supplementary Information

Below is the link to the electronic supplementary material.

Online Resource 1

Online Resource 2

Online Resource 3

Online Resource 4

Online Resource 5

Online Resource 6

Online Resource 7

## Data Availability

The data analyzed in this study from the Norwegian Brain and Spinal Cord Tumor Registry can be obtained upon reasonable request by application to helsedata.no. Further information is available from the first author upon request via Cassia.Bree.Trewin-Nybraten@fhi.no.

## Code availability

The code used to generate the results that are central to the paper’s conclusions is available at https://github.com/CBTrewin-Nybraten/regional-differences-in-glioblastoma-survival.
