# Supplementary material for: Unveiling regional differences in glioblastoma patient survival with real-world data from the Norwegian brain tumor quality registry
Source: J Neurooncol. 2025 Sep 11;175(3):1355–66. doi: 10.1007/s11060-025-05218-3 (PMC12511213; doi:10.1007/s11060-025-05218-3)
Supplement: Supplementary file 3 — Online Resource 3 [file 11060_2025_5218_MOESM3_ESM.pdf]

Online Resource 3

Kaplan-Meier overall survival by quartile (Q) of distance from patient’s residential municipality to the closest neurosurgical hospital and radiotherapy center. Adults diagnosed with histologically confirmed glioblastoma at 18–89 years during 2019–2023 (N = 1158).

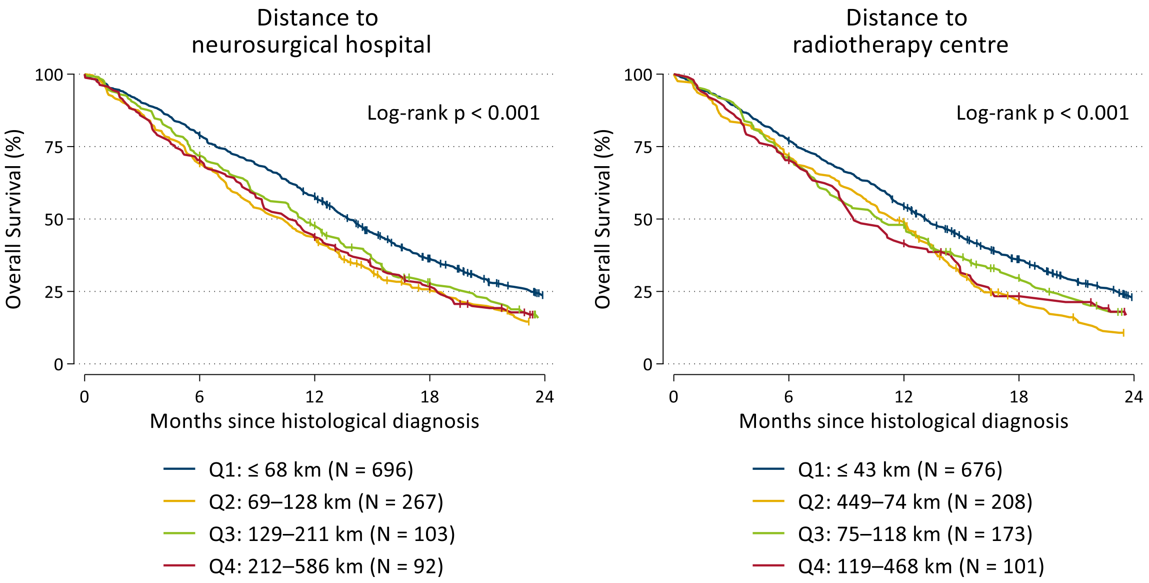

Article title: Unveiling regional differences in glioblastoma patient survival with real-world data from the Norwegian brain tumor quality registry

Journal name: Journal of Neuro-Oncology

Author names: Cassia Bree Trewin-Nybråten[1], Paul Christopher Lambert[1,2], Kirsten Marienhagen[3], Lasse Andreassen[4], Tom Børge Johannesen[1], Pitt Niehusmann[5,6], Leif Oltedal[7,8], Stephanie Schipmann[9,10], Anne Jarstein Skjulsvik[11,12], Ole Solheim[13,14], Tora Skeidsvoll Solheim[12,15], Terje Sundstrøm[8,9], Einar Osland Vik-Mo[16,17,18], Petter Brandal[18,19,20], Tor Ingebrigtsen[4,21], Erlend Skaga[16,17]

[1] Department of Registration, Cancer Registry of Norway, Norwegian Institute of Public Health, Oslo, Norway (CBT, PCL, TBJ)

[2] Department of Medical Epidemiology and Biostatistics, Karolinska Institutet, Stockholm, Sweden (PCL)

[3] Department of Oncology, University Hospital of North Norway, Tromsø, Norway (KM)

[4] Department of Neurosurgery, Otorhinolaryngology and Ophthalmology, University Hospital of North Norway, Tromsø, Norway (LA, TI)

[5] Department of Pathology, Oslo University Hospital, Oslo, Norway (PN)

[6] Division for Cancer Medicine, Oslo University Hospital, Oslo, Norway (PN)

[7] Mohn Medical Imaging and Visualization Centre, Department of Radiology, Haukeland (LO) University Hospital, Bergen, Norway

[8] Department of Clinical Medicine, University of Bergen, Bergen, Norway (LO, TS)

[9] Department of Neurosurgery, Haukeland University Hospital, Bergen, Norway (SS, TS)

[10] Department of Neurosurgery, University Hospital Muenster, Germany (SS)

[11] Department of Pathology, St. Olavs Hospital, Trondheim University Hospital, Trondheim, Norway (AJS)

[12] Department of Clinical and Molecular Medicine, Faculty of Medicine and Health Sciences, Norwegian University of Science and Technology, Trondheim, Norway (AJS, TSS)

[13] Department of Neurosurgery, St. Olavs University Hospital, Trondheim, Norway (OS)

[14] Department of Neuromedicine and Movement Science, Norwegian University of Science and Technology, Trondheim, Norway (OS)

[15] Cancer Clinic, St. Olavs University Hospital, Norway (TSS)

[16] Vilhelm Magnus Laboratory for Neurosurgical Research, Oslo University Hospital, Oslo, Norway (EOVM, ES)

[17] Department of Neurosurgery, Oslo University Hospital, Oslo, Norway (EOVM, ES)

[18] Institute for Clinical Medicine, Faculty of Medicine, University of Oslo, Oslo, Norway (EOVM, PB)

[19] Department of Oncology, Division of Cancer Medicine, Oslo University Hospital, Oslo, Norway (PB)

[20] Institute for Cancer Genetics and Informatics, Oslo University Hospital, Oslo, Norway (PB)

[21] Department of Clinical Medicine, Faculty of Health Sciences, UiT the Arctic University of Norway, Tromsø, Norway (TI)

Corresponding author: tor.ingebrigtsen@uit.no
