# Supplementary material for: Unveiling regional differences in glioblastoma patient survival with real-world data from the Norwegian brain tumor quality registry
Source: J Neurooncol. 2025 Sep 11;175(3):1355–66. doi: 10.1007/s11060-025-05218-3 (PMC12511213; doi:10.1007/s11060-025-05218-3)
Supplement: Supplementary file 5 — Online Resource 5 [file 11060_2025_5218_MOESM5_ESM.pdf]

Online Resource 5

Ninety-day postoperative mortality, by age at diagnosis and region. Adults diagnosed with histologically confirmed glioblastoma at 18–89 years during 2019–2023 (N = 1158).

	Ninety-day postoperative mortality, % (95% CI)		
**Region**	**Total**	**18 – 70 years**	**71 – 89 years**
South-East	12.1 (9.8 – 14.9)	6.6 (4.5–9.4)	23.0 (17.8–29.3)
West	8.7 (5.8 – 13.1)	7.5 (4.4–12.5)	12.1 (6.3–22.8)
Mid	7.8 (4.6–13.0)	7.8 (4.2–14.5)	7.7 (3.0–19.2)
North	20.7 (14.4–29.3)	9.0 (4.1–18.9)	36.7 (25.0–51.8)

Article title: Unveiling regional differences in glioblastoma patient survival with real-world data from the Norwegian brain tumor quality registry

Journal name: Journal of Neuro-Oncology

Author names: Cassia Bree Trewin-Nybråten[1], Paul Christopher Lambert[1,2], Kirsten Marienhagen[3], Lasse Andreassen[4], Tom Børge Johannesen[1], Pitt Niehusmann[5,6], Leif Oltedal[7,8], Stephanie Schipmann[9,10], Anne Jarstein Skjulsvik[11,12], Ole Solheim[13,14], Tora Skeidsvoll Solheim[12,15], Terje Sundstrøm[8,9], Einar Osland Vik-Mo[16,17,18], Petter Brandal[18,19,20], Tor Ingebrigtsen[4,21], Erlend Skaga[16,17]

[1] Department of Registration, Cancer Registry of Norway, Norwegian Institute of Public Health, Oslo, Norway (CBT, PCL, TBJ)

[2] Department of Medical Epidemiology and Biostatistics, Karolinska Institutet, Stockholm, Sweden (PCL)

[3] Department of Oncology, University Hospital of North Norway, Tromsø, Norway (KM)

[4] Department of Neurosurgery, Otorhinolaryngology and Ophthalmology, University Hospital of North Norway, Tromsø, Norway (LA, TI)

[5] Department of Pathology, Oslo University Hospital, Oslo, Norway (PN)

[6] Division for Cancer Medicine, Oslo University Hospital, Oslo, Norway (PN)

[7] Mohn Medical Imaging and Visualization Centre, Department of Radiology, Haukeland (LO) University Hospital, Bergen, Norway

[8] Department of Clinical Medicine, University of Bergen, Bergen, Norway (LO, TS)

[9] Department of Neurosurgery, Haukeland University Hospital, Bergen, Norway (SS, TS)

[10] Department of Neurosurgery, University Hospital Muenster, Germany (SS)

[11] Department of Pathology, St. Olavs Hospital, Trondheim University Hospital, Trondheim, Norway (AJS)

[12] Department of Clinical and Molecular Medicine, Faculty of Medicine and Health Sciences, Norwegian University of Science and Technology, Trondheim, Norway (AJS, TSS)

[13] Department of Neurosurgery, St. Olavs University Hospital, Trondheim, Norway (OS)

[14] Department of Neuromedicine and Movement Science, Norwegian University of Science and Technology, Trondheim, Norway (OS)

[15] Cancer Clinic, St. Olavs University Hospital, Norway (TSS)

[16] Vilhelm Magnus Laboratory for Neurosurgical Research, Oslo University Hospital, Oslo, Norway (EOVM, ES)

[17] Department of Neurosurgery, Oslo University Hospital, Oslo, Norway (EOVM, ES)

[18] Institute for Clinical Medicine, Faculty of Medicine, University of Oslo, Oslo, Norway (EOVM, PB)

[19] Department of Oncology, Division of Cancer Medicine, Oslo University Hospital, Oslo, Norway (PB)

[20] Institute for Cancer Genetics and Informatics, Oslo University Hospital, Oslo, Norway (PB)

[21] Department of Clinical Medicine, Faculty of Health Sciences, UiT the Arctic University of Norway, Tromsø, Norway (TI)

Corresponding author: tor.ingebrigtsen@uit.no
